# Hypercalcemia of Malignancy

**Published:** 2015-11-01

**Authors:** Steve Malangone, Christopher J. Campen

**Affiliations:** University of Arizona Cancer Center, Tucson, Arizona; Greenville Health System, Greenville, South Carolina

## Abstract

**CASE STUDY**:

A 60-year-old man initially presented with pain in the right upper quadrant in October 2010. A computed tomography (CT) scan of the abdomen pelvis completed at that time showed a mass at the junction of the body and tail of the pancreas and multiple large liver lesions. A CT-guided liver biopsy revealed low-grade neuroendocrine carcinoma.

The patient was initially started on systemic treatment with sunitinib (Sutent) and octreotide. He developed intolerable side effects, including nausea and migraine. Therapy was discontinued in October 2011, when a CT scan revealed evidence of disease progression. At this point, he was transitioned to everolimus (Afinitor). He was treated with everolimus, with overall stable disease, until a magnetic resonance image (MRI) of the abdomen and pelvis showed enlarging hepatic metastases in April 2014. Everolimus was discontinued.

The patient presented to the clinic to start third-line systemic therapy; he described the recent onset of disorientation at home, with difficulty in concentration and mild muscle weakness. He was found to be lethargic on the day of the visit. He was noted to have a 4-kg weight loss. Blood pressure was 82/45 mm Hg, with a heart rate of 115 beats/minute.

Lab tests revealed a serum calcium level of 12.7 mg/dL (9.5 mg/dL prior). At that time, the serum albumin level was 2.4 mg/dL. The corrected calcium for albumin was 14 mg/dL. The patient was treated with intravenous (IV) hydration, and vital signs normalized post treatment. Labs revealed an improvement in serum calcium to 11.8 mg/dL (corrected = 13.1 mg/dL). Additional laboratory analysis revealed vitamin D, 25-hydroxy level of 40 ng/mL (reference range, 20–50 ng/mL), parathyroid hormone–related protein of 5.2 pmol/L (reference range, < 2.0 pmol/L), thyroid-stimulating hormone of 1.04 mIU/mL (reference range, 0.35–4.00 mIU/mL). An electrocardiogram revealed sinus tachycardia with a QT of 31.6 ms (QTc of 38.4 ms). The patient improved symptomatically and was sent home.

The patient returned for repeat labs 1 week later, with worsening of previously described weakness and lethargy. The serum calcium level had increased to 13.2 mg/dL, with a serum albumin level of 2.8 mg/dL. Intravenous zoledronic acid (4 mg) was administered, and he was admitted for symptomatic hypercalcemia. He received continuous IV hydration with normal saline at 300 mL/hr and telemetry monitoring. Once hydrated, he was treated with IV furosemide. His serum calcium level rapidly improved to 10 mg/dL by day 2 of admission, and lethargy and weakness symptoms resolved. He was discharged from the hospital at that time.

After discharge, the patient continued on third-line capecitabine-based systemic therapy, with excellent radiologic and tumor marker response to therapy and monthly zoledronic acid infusion. Serum calcium levels returned to within the normal range and stable, and he remained without relapse of hypercalcemia. After 2 months, zoledronic acid was discontinued, and the serum corrected calcium remained within normal limits.

Hypercalcemia is a common, potentially life-threatening clinical syndrome associated with a variety of malignancies, including lymphomas as well as aerodigestive, uterine, endometrial, breast, neuroendocrine, cervical, and renal cell carcinomas. The historic incidence of hypercalcemia in the cancer population is noted to be as high as 20% to 30% and is associated with significant morbidity, including progressive cognitive dysfunction, dehydration, and acute renal failure (Stewart, 2005).

Hypercalcemia rates have declined significantly in recent years due to the prophylactic use of bisphosphonates. Uncorrected hypercalcemia may result in dysrhythmia, coma, and death, with an estimated risk of death as high as reports of as much as 50% mortality within 30 days of diagnosis (Ralston, Gallacher, Patel, Campbell, & Boyle, 1990). Appropriate diagnosis, evaluation, and management of hypercalcemia are important aspects of the advanced practice management of patients with malignancy.

## PRESENTATION

Patients with hypercalcemia can exhibit a wide spectrum of neurologic, gastrointestinal, renal, cardiac, and musculoskeletal signs and symptoms (see Table 1). The spectrum of symptoms of hypercalcemia tends to worse with the degree of hypercalcemia. Comorbities and rate of chance in serum calcium levels can impact the onset and severity of these symptoms. Patients with chronically elevated calcium are more likely to compensate than those with an acute onset of elevated serum calcium. Therefore, patients with hypercalcemia of malignancy are often symptomatic, given the relatively abrupt onset in patients with cancer. Elderly patients, in particular, are also more susceptible to neurologic symptoms from hypercalcemia than are younger individuals (Stewart, 2005).

**Table 1:**
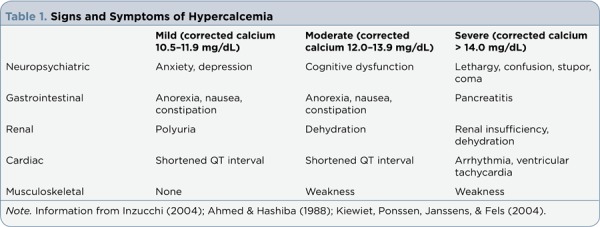
Signs and Symptoms of Hypercalcemia

Many of the symptoms of hypercalcemia are nonspecific and occur commonly due to other etiologies in patients with cancer. For example, anorexia, anxiety, dehydration, and weakness are all common symptoms for many individuals with cancer, as manifestations of the cancer itself or as adverse effects of antineoplastic therapy. The oncology advanced practitioner is challenged to be mindful of hypercalcemia as a possible differential diagnosis in patients with cancer who are experiencing these symptoms.

## ETIOLOGY

Hypercalcemia of malignancy can occur as a result of four different mechanisms, the most common of which is humoral hypercalcemia of malignancy (HHM). Humoral hypercalcemia of malignancy is caused by the ectopic production of parathyroid hormone–related peptide (PTHrP) by tumor cells. The PTHrP binds to parathyroid hormone receptors, inhibiting the action of osteoblasts and stimulating osteoclasts in the bone. The PTHrP also promotes renal tubular calcium reabsorption, which further elevates serum calcium by inhibiting elimination (Ratcliffe, Hutchesson, Bundred, & Ratcliffe, 1992).

The second most common mechanism associated with hypercalcemia of malignancy is osteolytic hypercalcemia. In patients with osseous metastases, increased cytokine activity in the region of lytic osseous metastatic lesions stimulates the activity of osteoclasts in the bone. When compensatory mechanisms are exceeded, the serum calcium level rises as a result, causing hypercalcemia (Francini et al., 1993; Clines & Guise, 2005). This is more common in multiple myeloma and solid tumors and less common in leukemias and lymphomas with bony involvement.

In the setting of lymphoma, malignant lymphocytes produce ectopic calcitriol (1,25-dihydroxyvitamin D), which leads to increased intestinal absorption of calcium. Normally, calcidiol (25-hydroxyvitamin D) is converted to calcitriol (1,25-dihydroxyvitamin D; i.e., active vitamin D) in the kidneys under the stimulation of PTH. Calcitriol increases intestinal absorption of calcium. When serum calcium levels rise, PTH is suppressed; this inhibits the conversion of calcidiol to calcitriol, thereby inhibiting intestinal absorption of calcium (Roodman, 1997). Some lymphomas (both Hodgkin and non-Hodgkin) produce ectopic calcitriol, thus bypassing and overriding the normal physiologic control of intestinal absorption of calcium, leading to increased serum calcium levels.

Finally, PTH may be produced ectopically by tumor cells. Elevated PTH increases osteoclastic activity, decreases osteoblastic activity, causes increased intestinal absorption of calcium, and decreases renal elimination. Tumors that secrete PTH are extremely rare and exist primarily in case reports of various solid tumors in the literature (Inzucchi, 2004).

## EVALUATION

Perhaps the most important component of evaluation is identification, as hypercalcemia can often be overlooked. The majority of calcium is bound to albumin. Free or ionized calcium is what is biologically active. Therefore, patients with low albumin levels and normal serum calcium levels actually may be hypercalcemic. Therefore, it is necessary to use a corrected calcium (CRC) to accurately identify hypercalcemia (CRC = serum calcium + 0.8 [4 - serum albumin]).

Serum CRC levels are not always precise and reliable, particularly in malignancies such as myeloma, which can produce additional calcium-binding proteins, thus overestimating the actual free calcium level (Ladenson, Lewis, McDonald, Slatopolsky, & Boyd, 1978). Ionized calcium levels provide more precise measures of serum calcium.

Further evaluation is directed at identifying an underlying cause to direct the proper plan of care. It is important for the advanced practitioner also to rule out nonmalignant causes of elevation of these levels. They include primary hyperparathyroidism and administration of thiazide diuretics, which may enhance renal tubular reabsorption of calcium.

**Figure 1:**
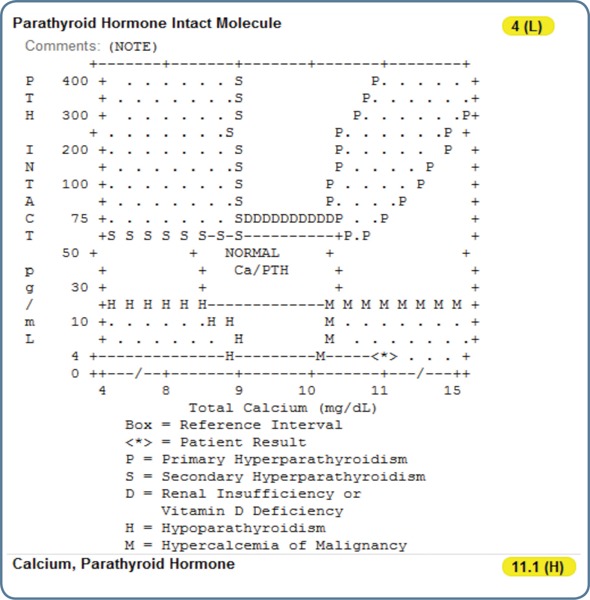
When serum calcium and parathyroid hormone (PTH) are checked at the same time, the relationship between these levels is expressed in this graph in most clinical laboratories. If both PTH and calcium are elevated, this suggests primary hyperparathyroidism. If PTH is high and calcium is low, this suggests secondary hyperparathyroidism. If PTH is low and serum calcium is low, this is indicative of hypoparathyroidism. In hypercalcemia of malignancy, PTH is typically appropriately suppressed in the setting of high serum calcium .

**Figure 2:**
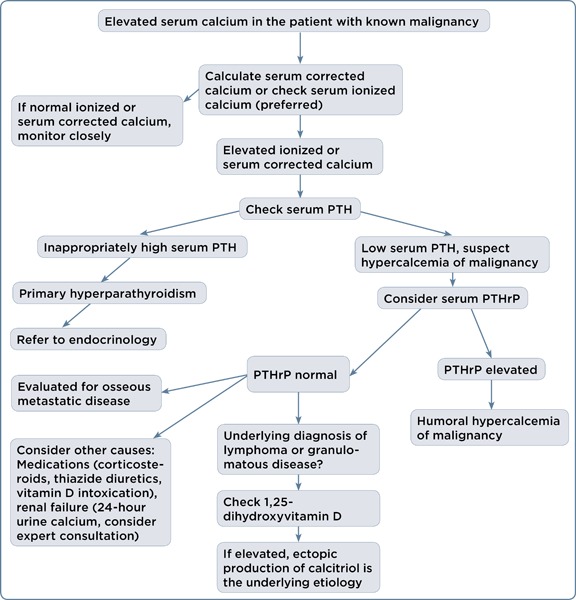
Diagnostic algorithm of hypercalcemia in the oncology setting. PTH = parathyroid hormone; PTHrP = parathyroid hormone-related peptide.

Evaluation should include intact PTH, which is typically suppressed in hypercalcemia of malignancy but elevated in primary hyperparathyroidism. Most laboratories will provide test results on a graph, which can be helpful in clarifying the etiology (Figure 1). In patients with lymphoma, plasma 1,25-dihydroxyvitamin D levels should be measured, as they will be elevated in 1,25-dihydroxyvitamin D syndrome (Stewart, 2005). See the algorithm in Figure 2 for an overview of diagnosing hypercalcemia in the oncology setting.

## TREATMENT

Treatment of hypercalcemia includes measures to reduce rapidly serum calcium levels, restore hydration status, and maintain durable control. Goals of management include rapid reduction in serum calcium levels, supportive care, and correction of the underlying cause.

Treatment of hypercalcemia of malignancy typically requires assessment of the severity of symptoms and the underlying cause. In more severe cases, or in patients with complex comorbidities, hospitalization is recommended. More stable patients with low-grade hypercalcemia may be treated in an outpatient cancer clinic.

Aggressive hydration followed by administration of IV furosemide is helpful in causing rapid reduction in serum calcium levels. Intravenous fluids should be given at a high rate, 200 to 500 mL/hr, as tolerated. Patients with preexisting renal dysfunction and congestive heart failure may require a more conservative rate of administration. All patients should be monitored closely, and patients should be assessed for signs and symptoms of fluid volume overload. After adequate hydration has been achieved, administration of IV furosemide causes calciuresis, which can quickly reduce serum calcium levels.

Intravenous bisphosphonate therapy has become the mainstay of treatment to promote a more durable response and should be given as soon as possible. Bisphosphonates work by blocking osteoclast activity and decreasing bone turnover. However, bisphosphonates do not reach maximum effectiveness until days 2 through 4, highlighting the importance of proper hydration (Rodan & Fleisch, 1996).

Data support the use of both pamidronate and zoledronate, with one study showing a slightly statistically significant superiority with zoledronate (Major et al., 2001; see Table 2). If the patient has moderate or severe hypercalcemia and a rapid reduction in calcium levels is beneficial, calcitonin should also be added. Calcitonin works by inhibiting renal tubular calcium reabsorption and blocks osteoclast activity. It has a rapid onset, but tachyphylaxis reduces the durability and limits its continued use (Ralston, 1992).

**Table 2:**
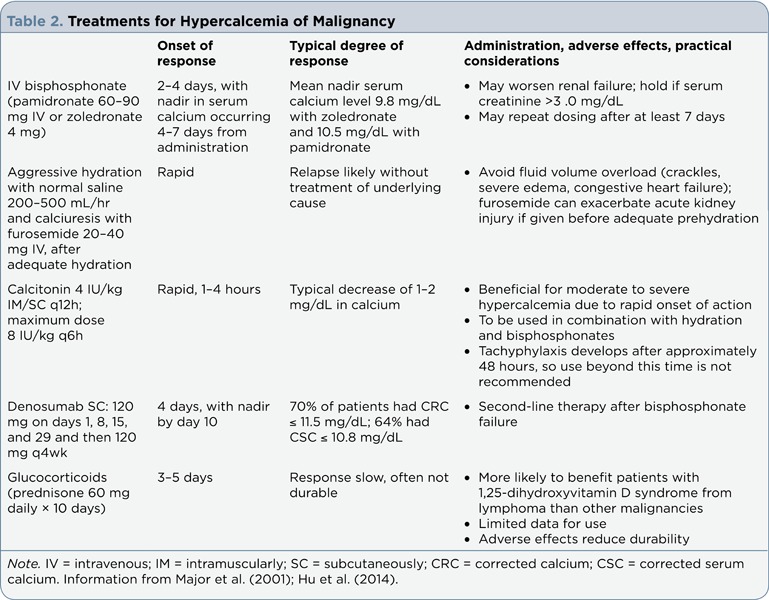
Treatments for Hypercalcemia of Malignancy

Patients who are still hypercalcemic by day 8 may require an additional dose of zoledronic acid. If they are still unresponsive, an alternative agent should be considered. Denosumab (Xgeva) has been shown to be effective in patients with treatment failure after administration of IV bisphosphonates (Hu et al., 2014).

Corticosteroids are another option, but adverse effects prevent these medications from being used for extended periods. Patients with hypercalcemia as a result of 1,25-dihydroxyvitamin D syndrome from lymphoma are most likely to benefit from corticosteroids. Concurrent treatment of the underlying malignancy is ultimately the best way to prevent recurrent hypercalcemia.

In patients with a contraindication to aggressive IV hydration and IV bisphosphonates, dialysis can be an effective second-line option but should be reserved for patients expected to recover and who have a reasonable prognosis overall (Stewart, 2005). This approach is recommended for patients with a glomerular filtration rate of less than 10–20 mL/min or in patients with congestive heart failure, which prohibits aggressive IV fluid administration.

Supportive measures suggested to enhance the plan of care include reducing calcium intake by removing calcium from the diet. One important and often overlooked consideration is removal of calcium from any parenteral feeding the patient may be receiving. Patients are encouraged to engage in as much weight-bearing activity as possible to promote calcium resorption by the bones. Serum phosphorus should also be monitored, and hypophosphatemia should be corrected through phosphate supplementation.

## CONCLUSION

Hypercalcemia of malignancy is a common and potentially life-threatening complication experienced by patients with cancer. It is often a clinical syndrome associated with aggressive disease and may occur at the time of diagnosis or clinical progression of therapy. Correction of hypercalcemia does not improve long-term survival without effective antitumor therapy (Ralston et al., 1990). Although immediate interventions are important, measures to treat and control the underlying disease will result in the most durable response.

In our case report, the patient went on to receive monthly bisphosphonate therapy after initial correction of serum calcium levels. When serum chromogranin and radiographic evidence showed good response to antineoplastic therapy, bisphosphonate therapy was discontinued. The patient was free from relapse of hypercalcemia while in a state of adequate disease control. Prompt identification, evaluation, and management of hypercalcemia are essential skills for the advanced practitioner in oncology.
